# Biological and Acoustic Sex Differences in Rat Ultrasonic Vocalization

**DOI:** 10.3390/brainsci11040459

**Published:** 2021-04-04

**Authors:** Charles Lenell, Courtney K. Broadfoot, Nicole E. Schaen-Heacock, Michelle R. Ciucci

**Affiliations:** 1 Department of Surgery, University of Wisconsin Madison, Madison, WI 53792, USA; Lenell@wisc.edu (C.L.); ckuehn2@wisc.edu (C.K.B.); nschaen@wisc.edu (N.E.S.-H.); 2 Communicative Sciences and Disorders, New York University, New York, NY 10001, USA; 3 Department of Communication Sciences and Disorders, University of Wisconsin Madison, Madison, WI 53706, USA

**Keywords:** ultrasonic vocalization, larynx, female, male, sex differences

## Abstract

The rat model is a useful tool for understanding peripheral and central mechanisms of laryngeal biology. Rats produce ultrasonic vocalizations (USVs) that have communicative intent and are altered by experimental conditions such as social environment, stress, diet, drugs, age, and neurological diseases, validating the rat model’s utility for studying communication and related deficits. Sex differences are apparent in both the rat larynx and USV acoustics and are differentially affected by experimental conditions. Therefore, the purpose of this review paper is to highlight the known sex differences in rat USV production, acoustics, and laryngeal biology detailed in the literature across the lifespan.

## 1. Introduction

Both male and female rats produce ultrasonic vocalizations (USVs) in a variety of contexts that hold communicative intent [1,2,3,4,5,6,7]. Adult rat USVs can be categorized into two primary types based on affective state and mean frequencies: 1) alarm USVs which are produced during negative affective states with a mean frequency near 22 kHz, and 2) 50 kHz USVs which are produced during positive affective states [1,6,8,9,10]. Pups produce USVs with an average frequency of 40 kHz to receive care from their dam (female parent) [8,9,11]. Sexual dimorphism is apparent in all three major categories of USVs. Therefore, the purpose of this review paper is to highlight the known sex differences in rat USV production and acoustics as well as laryngeal biological differences between sexes. All ages were included in this review of the literature. This review is focused on sexual dimorphism of the rat larynx and USVs; however, sex differences exist in other rodent species’ USVs (e.g., hamsters [12] and mice [13,14,15,16]) and sexual dimorphism of USVs is also mediated by sex differences within the central nervous system, not just the larynx [17,18,19]. Nevertheless, the rat model is widely used to study vocal communication in a variety of contexts such as social environment [1,5,7,9,20,21,22,23], neurogenic disorders [24,25,26,27,28,29], aging [30,31,32,33,34,35], and pharmacology [36,37,38,39,40,41,42,43,44], justifying the need for a comprehensive review of the literature attuned to sex differences.

## 2. Review of Sex Differences

### 2.1. Sexual Dimorphism of the Vocal Fold

Rat USVs are produced using a complex orchestration of the respiratory, laryngeal, and resonatory systems [45]. The whistle-like vocalization is produced by airflow passing through glottal and supraglottal spaces, and the configuration of these spaces can be altered by subglottic pressure and intrinsic laryngeal muscle activity [45,46,47,48]. Laryngeal motor innervation is primarily by the nucleus ambiguus through two divisions of the vagus nerve: superior and recurrent laryngeal nerves [49,50,51,52,53]. Intrinsic laryngeal muscles such as the cricothyroid and thyroarytenoid elongate the glottis and shorten/close the vocal folds to regulate the glottal geometry for specific USV types [46]. Several studies have demonstrated that vocal fold approximation/configuration is critical for production and modulation of rat USVs, making rat vocal folds a targeted investigation in voice-related research [46,47,48,54,55,56]. Additionally, rat vocal folds, like human vocal folds, are composed of a body (thyroarytenoid muscles) and cover (lamina propria, macula flavae, and epithelium) [57,58] and can produce audible vocalizations with vocal fold vibration in the frequency range 1–6 kHz [45,59,60]. However, because conspecific communication occurs by USV rather than audible vocalizations, this paper discusses USV only.

**Figure 1:**
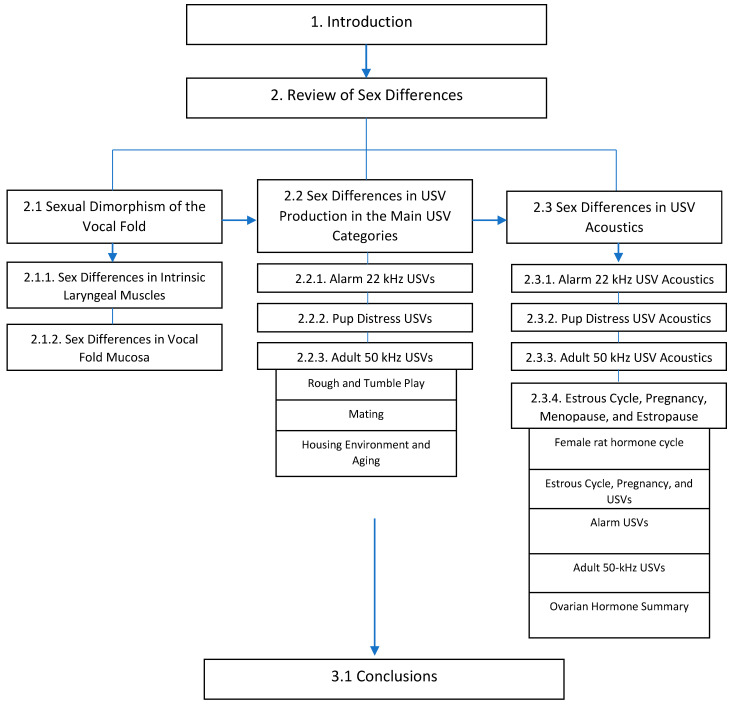
Organization of the manuscript.

**Figure 2:**
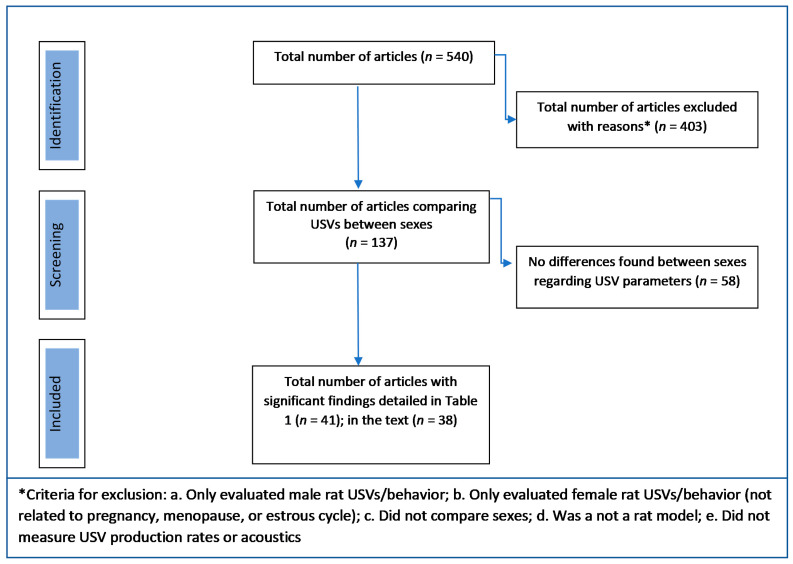
Number of articles reviewed and included in this article regarding sex differences in the rat USV with a PubMed search.

Understanding how hormones affect vocal structure and function (USV) is crucial for advancing science and clinical practice. As such, this paper reviews sex differences in laryngeal biology, USV production rates, and USV acoustics (Figure 1). More specifically, the summary of the known sex differences in the rat larynx was organized into intrinsic laryngeal muscles and vocal fold mucosa, and sexual dimorphism of the USV production and acoustics sections were summarized by primary USV category with rat strain and age reported as needed (Figure 1). To ensure that this review encompassed pertinent articles reporting sex differences in rat USVs, we created a PubMed search using the following search terms: ((USV) OR (ultrasonic vocalization) AND (female)) NOT (mouse). Authors then read the methods of the 540 article results and included all articles that compared rat USVs between sexes with significant findings within the results. The majority of articles were excluded for the following reasons: did not evaluate rat USVs, did not compare sexes, and did not include both sexes (Figure 2).

#### 2.1.1. Sex Differences in Intrinsic Laryngeal Muscles

Intrinsic laryngeal muscles are necessary to produce USVs, but few studies have examined sexual dimorphism within rat intrinsic laryngeal muscles [61]. Existing studies have primarily focused on evaluating the thyroarytenoid (TA) muscles, the primary muscles of the vocal folds [61,62]. The myofiber types of the lateral thyroarytenoid (LTA) and medial thyroarytenoid (MTA) muscles are similar between sexes, but the overall muscle areas of the LTA and MTA are larger in male rats [61]. Additionally, the individual minimum feret diameter of the myofibers of the LTA muscle are also larger in male rats [61]. Studies have not investigated sex differences in muscle fiber types and sizes in the other intrinsic laryngeal muscles such as the superior cricoarytenoid, lateral cricoarytenoid, posterior cricoarytenoid, cricothyroid, and alar muscles. Therefore, a lack of information exists regarding the potential sexual dimorphism of intrinsic laryngeal muscles.

Neuromuscular junctions (NMJs) of the TA muscles are also uniquely sexually dimorphic [62]. Female rats have more acetylcholine receptor fragments in the NMJs of the TA muscles but not the other intrinsic laryngeal muscles [62]. The study’s authors hypothesized that this sexually dimorphic NMJ feature would result in higher synaptic strength and was likely mediated by higher estrogen levels of female rats [62]. However, a recent study did not find NMJ morphological differences between ovariectomized (elimination of ovarian hormones) and control female rats, suggesting that the NMJ of the TA muscle may not be ovarian hormone dependent, and that sex differences in the TA muscles may be more likely influenced by male hormones (androgens) rather than female hormones such as estrogens [63].

Because few investigations have evaluated sexually dimorphic neuromuscular parameters of the laryngeal mechanism and even fewer studies have evaluated the effects of sex hormones on these parameters, how sex differences in the underlying neuromuscular laryngeal mechanisms influence acoustic differences in USV is unknown. Therefore, future studies characterizing the extent of sex differences and influence of sex hormones on the neuromuscular proprieties of the laryngeal mechanism are warranted.

#### 2.1.2. Sex Differences in Vocal Fold Mucosa

Both male and female rats have sex hormone receptors within the vocal folds, indicating that sex hormones bind within the vocal fold and potentially modulate physiological effects [64]. In general, rat studies have demonstrated that ovarian hormones more drastically affect properties of the vocal fold mucosa than androgens.

In female rats, sex hormones are critical to homeostasis of the pre-menopausal vocal fold mucosal tissues [64,65,66]. Several studies have demonstrated that removal of the ovaries (elimination of ovarian hormones) results in the remodeling of the vocal fold mucosa including the following: decreased cellular layers of the epithelium, increased edema of the lamina propria, and decreased collagen I, hyaluronic acid, and elastin of the lamina propria [64,65,66]. Although ovariectomy procedures have demonstrated vocal fold mucosal remodeling, orchiectomy procedures in male rats have not resulted in significant remodeling [64]. Therefore, the vocal fold mucosa appears to be differentially regulated between sexes with female rats having hormone-dependent mucosa, whereas the vocal fold mucosa of male rats does not seem to be affected by hormone status.

Pregnancy has also been shown to affect female rat vocal fold mucosa [67,68]. Pregnancy has been associated with the following histological changes in the vocal fold mucosa: increased edema, increased glycosaminoglycans, mast cell emergence, and increased cellularity of the lamina propria [67,68]. In addition, pregnancy has been found to change biomolecules within the vocal fold mucosa [67]. Specifically, pregnant rats had lower expression of nuclear factor-kappa B (a protein transcription factor related to immune response) and higher expression of mucin 5 subtype AC (the major mucin of the upper airway) [67]. Furthermore, progesterone levels were negatively correlated to the nuclear factor-kappa B, but estradiol levels were not correlated to either biomolecule [67]. Therefore, progesterone may activate transcriptional signaling responsible for mucosal changes during pregnancy and may consequently affect USV acoustics.

### 2.2. Sex Differences in USV Production in the Main USV Categories

**Figure 3:**
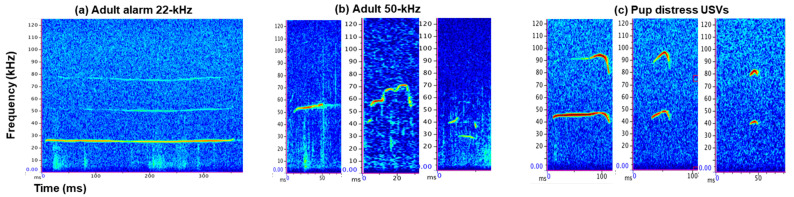
Spectrograms of the three main categories of rat USVs: adult alarm 22 kHz (**a**), adult 50 kHz (**b**), and pup distress USVs (**c**). We have included three subtypes of 50 kHz USVs (**b**) and three subtypes of pup distress USVs (**c**).

Rat USVs can be broadly categorized into three main categories: adult alarm 22 kHz, adult 50 kHz, and pup distress USVs (Figure 3). Within these broad categorizes, USV production rates are different between sexes. The following section will describe the sex differences in production of these USV types and subsequent sections will focus more specifically on acoustic differences.

#### 2.2.1. Alarm 22 kHz USVs

Alarm 22 kHz USVs are vocalizations produced during negative affective states with long durations (0.5–3.0 s), low frequencies (~22 kHz), and narrow bandwidths (1–4 kHz) [6,69]. Further, Blanchard identified six subtypes of alarm USVs during the presence of a predator: horizontal, linear ascending, linear descending, U-shaped, negatively accelerated ascending, and negatively accelerated descending vocalizations [70]. Sex differences in alarm 22 kHz USVs have been evaluated in response to both predators (a live cat) and laboratory experimental stressors [69]. In 2018, Inagaki reviewed sex differences in rat alarm 22 kHz USVs [69]. To complement this review, we will summarize the thematic sex differences in 22 kHz USVs incorporating more recent literature.

In general, female rats produce longer overall duration of alarm USVs in response to predators but shorter overall duration of alarm USVs in response to experimental stressors [69]; however, production of alarm USVs are influenced by both strain and sex [71].

For example, in response to fear conditioning training, Long–Evans female rats produced greater overall duration of alarm 22 kHz USVs than male Long–Evans rats; however, the opposite was true for Sprague–Dawley rats [71]. Additionally, following fear conditioning training, Sprague–Dawley male rats produced more alarm USVs than female Sprague–Dawley rats during contextual and auditory conditioned stimuli, whereas male and female Long–Evans rats had similar alarm USV productions in response to conditioned stimuli [71]. Likewise, another study that evaluated the effects of serotonin transporter deficiency in a fear condition found that female rats produced fewer alarm USVs than male rats [72].

Within strain, rats can be categorized as high vocalizing or low vocalizing, [73] and stressors have been found to differentially affect sexes of high- and low-vocalizing rats. Wistar rats exposed to chronic experimental stressors (variable lights, small cages, tail pinch, etc.) produced significantly increased levels of 22 kHz USVs for low-vocalizing male rats and high-vocalizing female rats [74]. Therefore, strain, sex, and vocalizing category all contribute to differences in alarm USV rates for rats.

Early life stress also has been shown to differentially impact alarm USV productions between sexes. A study that evaluated the effects of brief and prolonged maternal separation demonstrated that brief maternal separation attenuated fear conditioning (reduced alarm USV production and freezing behavior) in both male and female Sprague–Dawley rats; however, in general, male rats produced greater overall duration of alarm USVs than female rats during fear conditioning [75]. Neonatal maternal separation resulted in changes to 22 kHz USV production in adulthood, with fewer 22kHz USVs in response to a stressor for female Sprague–Dawley rats but more 22 kHz USVs in Sprague–Dawley males, demonstrating an opposite-sex effect [76,77].

Playback of alarm USVs to adult rats also affects behavior differently between sexes. A playback of 22 kHz alarm USVs resulted in more long-lasting behavioral inhibition in female rats than male rats [78]. This finding highlights that although rats may have hormone-mediated, sexually dimorphic USV rates and different acoustic characteristics, these differences may not be directly related to laryngeal differences but rather differences in behavior mediated by the central nervous system.

Hormones likely contribute to the sexual dimorphism observed in alarm USV production and differentially affect alarm USV productions. For example, in response to an air puff, female Wistar rats produce shorter overall duration of alarm USVs than males on both proestrus and diestrus phases of the estrous cycle [79]. This difference was hypothesized to be due to testosterone level differences between sexes and tested in subsequent experiments. In response to an air puff, castrated male Wistar rats produced shorter overall duration of alarm USVs than sham-operated or castrated male rats with a testosterone implant [80]. Additionally, because other anxiety responses (freezing and defecation) were not reduced in the castrated male rats, the lower alarm USV emissions did not indicate a reduced startle response [80]. The reduced emission rate is more likely indicative of a reduction in dominant behaviors [80]. Nevertheless, in response to an air puff, alarm USV production of ovariectomized female Wistar rats did not differ between female rats with testosterone implants and female rats with cholesterol implants [79]. Furthermore, alarm USV productions were similar between female rats in diestrus 1 (low hormones) and proestrus (high hormones), indicating no role of ovarian hormones in alarm USV productions. Therefore, while male alarm USVs may be influenced by sex hormones, female alarm USVs may not be. Nevertheless, these results were found in one anxiety context with one strain of rat and should not be assumed to be identical in all anxiety contexts or all rat strains.

Taken together, these results demonstrate that fearful conditions differentially affect male and female rat behavior including their production of alarm USVs. Nevertheless, more research is warranted to evaluate the patterns of hormonal influence across strain and sexes before definitive conclusions can be made regarding sexual dimorphism of alarm USV productions.

One uniquely male 22 kHz USV subtype is the post-ejaculation vocalization [81,82]. This extended vocalization is produced by the male approximately 30 seconds post-ejaculation and continues, repeatedly, for approximately 75% of the entire post-ejaculatory refractory period [81]. Such USVs are characterized as being highly frequency modulated, specifically within the medial terminal segments of the USV [83]. All spontaneous copulatory behavior ceases during this refractory period [84]. While such copulatory behaviors are associated with 22 kHz vocalizations, alarm USVs have also been observed in other aspects surrounding mating. For example, prior to mounting, male rats produce 22 kHz USVs as the rat approaches ejaculation, particularly if the female is non-receptive to male mounting and/or if the male was unsuccessful [81]. It is hypothesized, in the copulatory context, that this USV subtype represents a “de-arousal” mechanism or a type of “motivational cut-off” [85]. This may enforce mating separation between the male and female rats while still maintaining social contact [81]. Given the few studies available and the several-decade gap in published studies, more research into this unique vocalization is certainly warranted.

#### 2.2.2. Pup Distress USVs

Rat pups produce USVs ranging in mean frequency between 30 and 65 kHz when separated from their mother and associated litter. These USVs are generally referred to as distress USVs [36]. The duration of distress USVs is variable (ranging between average durations of 80 and 150 ms) and starts with very short USVs at younger ages with increasing duration with maturation [86]. The distress USVs are unique in that the timeframe in which pups produce them is relatively short (~18 days of age). While the increase in call rate may be correlated with a heightened state of anxiety upon separation, both duration and frequency may reflect developmental changes pre- and post-weaning [87]. Pups are completely reliant on the mother for survival prior to weaning, supporting the hypothesis that these distress USVs are produced in the context of separation/isolation, and are consequently important for pup survival [36,88].

The pup USVs have also been described as occurring in the frequency range 40–70 kHz, further being classified into 2 groups: 40 kHz/300 ms and 66 kHz/21 ms [88]. These classes have specific relationships with both respiration and behavior and are produced during pup movement [88]. While 40 kHz distress USVs have been observed in the context of isolation, they can also be elicited in a more naturalistic setting when mothers engage in rough handling with the pups [88]. In contrast, 66 kHz are not related to the behavioral conditions [88]. In terms of respiration, distress USVs alter the length of expiration, lasting for the entirety of the expiration cycle, whereas 66 kHz USVs do not alter the respiratory signal in pups when mild foot shocks were administered [88].

Pup distress USVs can also occur following a drop in ambient temperature [9]. This behavior is thought to relate to a pup’s dependence on the dam for survival, as pups cannot regulate their own body temperature. Additional research investigating the role of anxiogenic drugs on pup USVs supports the hypothesis that these USVs are correlated with presumptive distressed/anxious states, as administration of said drugs (e.g., selective serotonin reuptake inhibitors) lead to a reduction or complete blocking of the USVs [9,89,90,91]. Studies assessing selective breeding as well as breeding over several generations further support this hypothesis [92,93]. Pups bred to produce high rates of isolation-induced USVs for multiple generations demonstrated increased anxiety-related behaviors in adulthood. Additionally, rats who were selectively bred to demonstrate an anxious phenotype in adulthood produced more distress USVs as pups compared to a less anxious phenotype [92]. In summary, pups produce unique distress vocalizations prior to weaning that seem important to their survival and are influenced by strain and genetic lines.

In the context of sex differences, current research suggests preferential retrieval of male pups by the dam which may be attributed to sex differences in pup distress USVs [17]. Male pups produce significantly more distress USVs with a lower mean frequency and lower amplitude that results in preferential retrieval of the dam [17]. Research has shown from postnatal day (P)-2 to 3 through P-12 to 13, males pups tend to produce distress USVs more frequently than female pups, which results in the dam retrieving and returning male pups preferentially to female pups [94]. Therefore, sexual dimorphism of the pup distress USVs may result in the female rat prioritizing male pup survival.

Although few studies have investigated sex differences in distress USVs in typical/normal rat pups, many studies have investigated how drugs, neurological disorders, endocrine disruptors, diet, and environmental condition differentially affect male and female pup distress USVs. Table 1 summarizes major sex differences found in USV rate and acoustics for experimental models organized by model, age, and strain. While not all ages are prior to weaning (~P21), most summarized studies in this table measure USV rates and/or acoustics within this timeframe (Table 1). Because the sex differences are not uniform across studies or models, the articles are summarized individually. While this table highlights major USV sex differences present in experimental rat models (particularly rat pups), not all experiments find sex differences in USV production or acoustics.

#### 2.2.3. Adult 50 kHz USVs

Both male and female rats produce 50 kHz USVs during various social contexts such as rough-and-tumble play, mating, and in isolation. These USVs are critical to the communicative intent of the rats and often impact the behavior of conspecifics [134,135,136]. Because 50 kHz USVs are often observed in appetitive situations and during physical interactions, features of these USVs have been investigated in different social contexts. These 50 kHz USVs can be subcategorized based on spectral features such as duration and frequency modulation. Wright et al. introduced 14 categories of 50 kHz USVs [37]; however, many studies use simpler categorization such as flat vs frequency modulated [137]. Although there is no current consensus on rat 50 kHz USV subcategories, rats are known to produce a variety of these vocalizations, and recent advancements in the efficiency of USV data analysis will lend to greater cross-institutional collaboration to better elucidate USV subcategories and their communicative relevance [138]. Furthermore, these social USVs such as alarm and pup USVs are also influenced by rat strain [139].

##### Rough and Tumble Play

During social interactions, such as social play, both young and aged rats will frequently produce 50 kHz vocalizations [7,140,141]. These vocalizations are thought to be produced to promote playful contact with peer rats and function as play signals [22]. During rough-and-tumble play, 50 kHz USVs often co-occur with attack-like, play behaviors [23]. These interactions, however, can transition beyond play fighting and into more serious fighting behavior [142]. Specifically, when pairs of unfamiliar adult males were exposed to each other, there was an increased risk to escalate into aggressive behavior if one partner is devocalized, or unable to communicate with USVs [143]. Thus, the importance of communication during rough-and-tumble play is critical to prevent this escalation [22]. Therefore, vocal communication during rough-and-tumble play has been investigated to explore the social ecological value it provides. To further explore their utility, sex differences in USV production have been identified. In rough-and-tumble play, male rats produce a greater amount of 50 kHz USVs when compared to female rats [143]. This sex difference has been attributed to males desire to engage in more rough play [142], but further exploration is warranted.

##### Mating

In addition to play-based social interactions, 50 kHz USVs are produced in mating contexts to initiate approach behaviors of mating partners [4]. Research findings are mixed regarding the role of USVs in mating, but one theory suggests that male 50 kHz USVs are prosocial in nature and elicit female copulation behaviors [144,145,146,147]. Other results suggest that female USVs do not provide mating incentive for male counterparts and instead support that male rats will show sexual interest in the female rat regardless of the presence of USVs [148,149,150], and male USVs did not influence female USV production [146]. However, the presence of an estrus (sexually receptive) female rat significantly increases male vocalizations [151]. Additionally, female vocalization rates were significantly increased during peak periods of sexual receptivity (during estrus), in contrast with male-only vocalizations, which were not linked to sexual receptivity [152,153]. In regards to female USVs, it has been found that female rats produced higher proportion of frequency modulated USVs to male peers compared to female peers, and females produced a higher total number of USVs when in the presence of male rats that have not been castrated [154]. These findings suggest that female vocalizations are also influential in motivating sexual interactions and may indicate communicative intent during opposite-sex encounters.

##### Housing Environment and Aging

Both housing environment and age influence USV production rates and acoustics in male rats. Social isolation influences USV production rates and acoustics depending on the length of time of isolation. Wöhr et al. found that male rats exposed to a brief period of social isolation produced more than twice the amount of USVs when compared to other rats, likely due to increased social motivation after isolating [5]. However, after longer periods of isolation (2–6 months), studies have found that socially isolated male rats produced fewer 50 kHz USVs with lower amplitude in response to a female rat than socially-housed rats [155,156]. Thus, it appears that while short-term social isolation may increase USV production rates in social situations, long-term social isolation decreases the number and amplitude of social USVs. The inclusion of female rats in future studies is warranted to understand how sexes may respond differently to social isolation in adulthood.

In male rats, aging has been shown to change USV acoustics with older rats producing fewer 50 kHz USVs with reduced peak frequency, frequency bandwidth, and amplitude in a mating context [31,34,35]. These changes to USV acoustics co-occur with non-muscular and neuromuscular changes in the larynx. Some of these changes include the following: reduced hyaluronic acid, reduced elastin densities, and increased collagen densities of the vocal fold [35]; motorneuron loss of the nucleus ambiguus [34]; deinnervation-like changes to the neuromuscular junction of the thyroarytenoid muscles [31,157,158,159,160]; reductions in muscle-twitch functions of the thyroarytenoid muscles [161]; and alterations to intrinsic laryngeal myofiber structures [33,158,161,162]. Although many of these changes may contribute to functional age-related deficits observed in rat larynx, as previously mentioned, USV production is a complex orchestration of many muscular subsystems that which simultaneously under age-related changes and cannot be explained by a single muscular system such as the larynx. Importantly, most of the studies investigating age-related changes to the larynx have been completed solely with male rats prohibiting any insight to sex differences in the aging rat larynx.

### 2.3. Sex Differences in USV Acoustics

#### 2.3.1. Alarm 22 kHz USV Acoustics

Although studies have confirmed differences between male and female alarm USV productions, most studies have focused on solely the number or overall duration of alarm USVs produced in fear contexts and have overlooked acoustic features or alarm subtypes. In Blanchard’s anti-predator USV study, female rats produce more frequent alarm USVs with a higher mean frequency and shorter duration [70]. In the response to a predator, male rats primarily produced negatively accelerated descending frequency alarm USVs, whereas female rats primarily produced linear descending alarm USVs. Another study that evaluated the effects of serotonin transporter deficiency in a fear condition found that female rats produced fewer overall alarm USVs than male rats with a higher frequency modulation and longer USV duration than males [72]. This finding complemented other studies that found higher frequencies of 22 kHz alarm USVs of female rats [70,105,163,164].

In summary, although alarm emission rates have been revealed to be different between sexes, sex differences in the subtypes and acoustic parameters of alarm USVs are relatively unexplored. The sex differences may be differentially regulated by the endocrine system with male rat alarm USVs being affected by sex hormones and female rat alarm USVs having less hormone dependence.

#### 2.3.2. Pup Distress USV Acoustics

As previously described, pups produce distress USVs during approximately the first 3 weeks of life in response to separation/isolation from the dam. This distress signal functions as both a social and survival act and signals the dam to retrieve and care for the pup. Because male pups produce more distress USVs, and these USVs tend to be lower in both mean frequency and amplitude compared to female pups, dams tend to respond to the male pups producing these USVs more so than female pups [17,36]. This sexual dimorphism may be mediated by the *FOXP2* gene, with a general reduction in *FOXP2* protein observed in females compared to males [17]. Although few studies have investigated the sexual dimorphism of distress USVs of typical rat pups, several studies in Table 1 highlight sex differences in distress USVs in various experimental models.

#### 2.3.3. Adult 50 kHz USV Acoustics

While USV acoustic properties have not been extensively studied between sexes, there have been a small number of studies exploring the difference between male and female rat vocalizations in terms of specific acoustic parameters. One study explored the impact of social situations on vocalizations between sexes, which revealed that female rats produced a higher proportion of frequency modulated 50 kHz USVs when exposed to a male partner compared to a female partner [154]. This suggested that female USVs may be indicative of sexual motivation. Other studies found that during rough-and-tumble play, levels of 50 kHz vocalizations was decreased as a result of Cacna1c haploinsufficiency, a gene implicated in social signal processing, which was more robustly noted in males than females [78]. Additionally, studies have explored acoustical parameter differences in male and female rats in isolation. Specifically, during isolation, the mean frequency of 50 kHz USVs was significantly lower in males than in females [61]. While there is work to be carried out in acoustic analysis exploring sex differences, these studies highlight the need for the inclusion of male and female rats into experimental studies as we continue to learn how social settings impact vocalizations produced by both sexes.

#### 2.3.4. Estrous Cycle, Pregnancy, Menopause, and Estropause Effects on USV Acoustics

##### Female Rat Hormone Cycle

The female rat, like other mammals, has an ovarian hormone cycle that begins following sexual maturation (puberty); undergoes cyclical regulation by the hypothalamus, ovaries, and pituitary gland; is ceased during pregnancy; and finally undergoes age-related dysregulation and subsequently infertility [165]. Nevertheless, the estrous cycle and age-related changes in the rat are uniquely different from other mammals.

In brief, female rats reach sexual maturity at ~3 months of age [166]. The typical estrous cycle of a female rat lasts between 4 and 5 days beginning with proestrus, which is ~14 h and has both high estradiol and progesterone levels (female rat might be receptive during proestrus), estrus (the sexually receptive state) which is ~24–48 h and has low estradiol and progesterone levels, and finally diestrus states which hormone levels begin to rise and the female rat refuses copulation [166,167]. At ~9 months of age female rats will begin to experience irregular cycling for ~1 month and enter estropause that has three stages: constant estrus, persistent diestrus with irregular cycling, and persistent diestrus [165,168]. An important distinction of rat estropause is that rats continue to secrete low-moderate ovarian hormones during constant estrus and elevated ovarian hormone levels during persistent diestrus, which contrasts to humans who experience dramatic loss of ovarian hormones at menopause [165,168]. Because of this difference in ovarian hormone status, the effects of menopause are studied using ovariectomy procedures in the rat model, which more closely mimics menopause of humans by eliminating the primary production of ovarian hormones via removal of the ovaries.

The subsequent sections will summarize the known effects of the ovarian cycle of the female rat on USV production and acoustics. The scant knowledge will be evident in the review.

##### Estrous Cycle, Pregnancy, and USVs

Few studies have investigated the effects of the hormone cycle or pregnancy on USV acoustics. Furthermore, to our knowledge no studies to date have investigated the effects of estropause stages on USV production or acoustics. In the following sections, both the USV production rates and acoustics will be described according to USV type: alarm 22 kHz USVs and 50 kHz USVs.

##### Alarm USVs

Few studies have evaluated the effects of the estrous cycle on alarm 22 kHz USVs. One study evaluated the total duration of alarm USVs produced after a puff of air and found no differences between proestrus and diestrus stages [79]. Nevertheless, although the overall duration of alarm USVs did not differ between the two evaluated estrous states, the entire cycle was not evaluated, and this analysis did not include acoustics. Therefore, the effects of the estrous cycle on alarm USV production and acoustics are unknown.

Although several studies have evaluated how perinatal conditions affect USVs, the effects have primarily been evaluated in the pup offspring rather than the pregnant dams (Table 1). However, two studies have demonstrated that stress affects dam USVs [169,170]. In one study postpartum dams that received brief or long-term separation from their litters, produced more 22 kHz USVs in response to a startle stimulus than control dams [169]. In another study where the non-pregnant female cagemate of a pregnant dam underwent two 30-min stress tests for five consecutive days and then was recorded with pregnant female cagemate, the stressed non-pregnant females produced more 22 kHz USVs during interaction while the pregnant bystander produced more 50 kHz USVs than the stressed non-pregnant females [170]. Therefore, although perinatal models have demonstrated effects on pup USVs, perinatal effects can also affect dam USVs, and currently it is unclear if dam USVs influence the USVs of their pups revealing a large gap in the literature.

##### Adult 50 kHz USVs

In mating contexts, several studies have found that female Long–Evans rats produce more 50 kHz USVs during receptive (proestrus and estrus) stages of the estrous cycle [153,171,172]. Because high rates of 50 kHz USVs are observed at both high hormone (proestrus) and low hormone (estrus) stages, these studies have collectively suggested that 50 kHz USV production rate may serve as a proceptive cue to male rats in mating contexts rather than a hormonal effect [153,171,172].

In a mating context, the USV acoustics of Long–Evans female rats are influenced by the estrous cycle [153]. In general, USV frequency parameters (such as lowest, highest, and median frequencies) are highest during high hormonal states (diestrus II and proestrus) and lowest during low hormonal states (estrus and metestrus) [153]. USV intensity (power) tends to be greatest during low hormonal states (estrus and metestrus), whereas duration and USV complexity (frequency modulation) tend to be greatest during high hormone (diestrus II and proestrus) and receptive states (proestrus and estrus) [153]. Therefore, USV acoustics are influenced by copulation behavior and hormone levels in normal-cycling female rats.

Another study found that hormonal injections influenced USV acoustic parameters of trill and flat-trill 50 kHz USVs during clitoral stimulation [147]. In this study, rats were ovariectomized and treated with estradiol, progesterone, estradiol + progesterone, or a vehicle. The combined estradiol + progesterone treatment significantly increased the rate of USVs as well as the duration and complexity of the USVs [147]. This finding mirrors the previously mentioned study that found USV duration and complexity to be greatest during high hormone/receptive states.

In a mating context, an ovariectomy affects the rate of USV production but has minimal effects on USV acoustics when compared to USVs of normal-cycling rats. The elimination of the cycle via ovariectomy overall reduces the number of USVs produced during mating contexts [153,171,172]. Studies found that ovariectomized Long–Evans female rats produced fewer 50 kHz USVs in a mating context than receptive age-matched females [153,171,172]. Additionally, in ovariectomized rats the USV acoustic parameters of frequency, complexity, intensity, and duration did not differ from control rats when compared across the estrous cycle [153]. Nevertheless, this reduction in USV rate can be counteracted with estradiol + progesterone injections [151,173]; however, estradiol or progesterone alone does not increase the number of 50 kHz USVs in ovariectomized Long–Evans rats in mating contexts [173].

In a non-mating social context, ovariectomized Sprague–Dawley rats receiving estradiol produced fewer 50 kHz USVs than ovariectomized Sprague–Dawley rats without hormone treatment [174]. Although the study’s authors predicted a higher 50 kHz USV production rate in rats receiving estradiol treatment, the decreased USV production may be indicative of improved social memory of the estradiol treatment group. Additionally, combined estrogen and progesterone treatments may be required to enhance social USVs. In a similar non-mating social context, ovariectomized Long–Evans female rats produce a similar number of USVs with similar acoustics to age-matched normal-cycling rats [153]. These results indicate that the estrous cycle influences the rate and acoustics of 50 kHz USVs during mating contexts more than non-mating social contexts.

In social isolation, the estrous cycle has less influence on USV parameters [153]. In isolation, female rats in estrus produced the most USVs with the lowest frequency parameters, greatest intensity, and complexity [153]. Female rats in metestrus produced the USVs with the least complexity, and shortest duration [153]. These results indicate that both the behavior and hormonal levels of the ovarian cycle influence USV acoustics of the normal-cycling female rat.

##### Ovarian Hormone Summary

In summary, the effects of the estrous cycle and ovarian hormones require further study to determine their influence on the female rat USV. To date, in the normal-cycling female rat, the estrous cycle is known to influence the number and acoustics of the 50 kHz USV in social contexts (primarily mating contexts). Additionally, the ovariectomy is known to reduce the number of 50 kHz USVs produced during mating contexts but not the acoustic parameters. Collectively, studies demonstrate an effect of ovarian hormones on 50 kHz USVs.

## 3. Conclusions

Biological and acoustic sex differences are apparent in the rat laryngeal mechanism. The type and acoustic features of USVs are different between male and female rats and are dependent on age, strain, and experimental models. Additionally, rat vocal folds are also sexually dimorphic which may contribute to the observed USV production and acoustic sex differences. This sexual dimorphism has been partially attributed to sex hormones; however, few studies of the laryngeal mechanism have investigated the role of hormones in influencing USV production and acoustic features. With recent advances in technology (such as DeepSqueak [138]) which simplifies and reduces the time burden of USV analysis, sexual dimorphism of the rat larynx can be further explored.

## Figures and Tables

**Table 1:** Summary of sex differences found in USV acoustics for experimental models.

Model	Sub Model	Age	Strain	Recording Duration	Major Sex Difference(s) in USV Acoustics
Drug exposure	Prenatal cannabis	P10	Wistar	15 s	Male pups produced fewer distress USVs during isolation, whereas females did not [95].
	Prenatal alcohol	P40–42	Wistar	10 min	For males, high ethanol exposure resulted in more 22 kHz and fewer 50 kHz USVs during play, whereas ethanol exposure did not affect female USV production during play [96].
		~P38–P48	LE	12 min	Prenatal exposure to alcohol decreased the mean frequency and total duration of 50 kHz USVs during same-sex social interaction for male rats, but not female rats [97].
		P28 P42	LE	10 min	At P28, during play female control whisker clipped rats produced more 22 kHz USVs than other female groups. At P42, during play male rats overall had more 50 kHz USVs than female rats [98].
	Postnatal alcohol	P15	SD	6 min	Neonatal alcohol exposure significantly reduced distress USV rate for both sexes and significantly increased USV latency in female pups. Agmatine reduced these deficits, in female but not male pups [99].
		P25, P35, P110–P120	SD	45 min	Adult alcohol-treated males produced more 22 kHz USVs following initial handling which was suppressed with the startle stimulus than male rats receiving water. Alcohol did not affect female 22 kHz USV rate. Overall, male rats had a greater number of 22 kHz USVs in response to startle stimulus [100].
		~2–5 mo.	NS	4 h	Female rats produced more 50 kHz USVs than male rats during experimental conditions. EtOH males produce 50 kHz USVs with a higher mean frequency and greater power than EtOH females. EtOH males produced 22 kHz USVs with a lower mean frequency, reduced bandwidth, and longer duration than EtOH females [101].
	Cocaine	P90	SD	15 min	During foot shock procedure males had a dramatic increase in 22 kHz USVs and decrease in 50 kHz USVs. Male rats also had longer duration of 22 kHz USVs [102].
		P1, P14, P21	SD	5 min	At P1, both male and female pups with prenatal cocaine exposure (PCE) produced fewer distress USVs than saline or untreated pups and male pups with PCE produced fewer USVs with at least one observable harmonic than male saline or untreated pups. At P21, male PCE rats produced more USVs with longer overall total duration of USVs than female PCE rats [103].
		P10, P11	SD	5 min	Male pups produced more distress USVs than female pups during the final 2 min of a 5 min isolation test [104].
	Morphine	P130–P288	LE	45 min	In the presence of a cat, both male and female rats produced fewer 22 kHz USVs when exposed to morphine. Additionally, both control and morphine females produced significantly more 22 kHz USVs with longer total duration than male counterparts [105].
	Oxycodone	P3 P6 P9 P12	SD	3 min	Isolation distress USVs peaked in production rate at P9 for males and P6 and P9 for females [106].
	Fluoxetine	P6	SERT	3 min	Fluoxetine reduced the total duration of distress USVs for male pups but did not affect female USV total duration [107].
	Diazepam	P3–P18	Wistar	3 min	Overall, male pups in all experimental conditions produced more distress USVs than females [108].
	Trimethylolpropane phosphate (TMPP)	P8, P14	LE	1 min	Males with prenatal TMPP treatment produced more distress USVs than control males, control females, and TMPP females [109].
Neurological disorder models	Shank3 deficiency	P7	Shank3	3 min	Fewer distress USVs were observed in male Shank3 −/− pups but not females [110].
	Pax6	P7	rSey2/+	5 min	Female rSey2/+ rat pups produce fewer distress USV from wild-type female pups, which was not observed in male rat pups [111].
	Valproic acid	P9 P31–P32 P65–P70	SD	5 min 10 min	In general, female rats had shorter duration of 50 kHz USVs during isolation, same-sex play, and same-sex social interaction than male rats. Female rats also had fewer 50 kHz USVs in same-sex social interaction [112].
	Valproic acid, chlorpyrifos	P7	Wistar	3 min	In isolation, male pups produced more distress USVs [113].
	Valproic acid, poly(I:C)	P6	SD	3 min	In the poly(I:C) condition, male pups produced more distress USVs than females [114].
	Cacna1c	P32–P34	Cacna1c	5 min	For control animals, female rats produced fewer overall 50 kHz USVs during same-sex play, with fewer step USVs and more trill USVs, than males. Female rat USVs also had a higher peak frequency. For experimental animals, female rats produced a similar rate of 50 kHz USVs during play as male control animals, whereas experimental male animals had reduced 50 kHz USV production during play [115,116].
	MAM	P60	SD	10 min	During same-sex social interaction, MAM exposure decreased the total number of 50 kHz USVs and increased the percentage of short USVs and decreased the percentage of frequency-modulated USVs for both sexes. However, control females had fewer frequency modulated USVs than control males, whereas it was opposite for MAM groups [117].
		P8 P30 P31–P32	SD	3 min 3 min 10 min	At P8, males pups produced distress USVs with a lower frequency and reduced bandwidth than females. At P30, males produced tickle-induced 50 kHz USVs with a higher center frequency than females. At P31-P32, during same-sex play, males produced more USVs with greater bandwidth than females [118].
	AVP	P34 P44	Brattleboro	10 min	Males produced more trill 50 kHz USVs during same-sex play than females [119].
		P34–37	Brattleboro	10 min	Males produced more 50 kHz USVs than females during same-sex play [120].
	PD	2–8 mo	Pink1-/-	90 s	Pink-/- female rats did not have as many 50 kHz USV deficits as Pink1-/- male rats in a mating context [121].
	SE	P15 P16 P21	Wistar	5 min	SE male pups had a decrease in USV latency than control pups, which was not observed in female pups [122].
	Liposaccharide (LPS)	P11	Wistar	5 min	Prenatal LPS exposure caused male pups to produce fewer distress USVs, but this was not observed with female pups [123].
	Ischemic brain injury	P12	Wistar	3 min	Overall, ischemic pups produced fewer distress USVs than control pups with male ischemic pups experiencing greater reductions in USV call subcategories than female ischemic pups [25].
Endocrine disruption	A1221 VIN	P80–P100 d	SD	5 min	In a mating paradigm, VIN males produced fewer 50 kHz USVs than control males. A1221 produced 50 kHz USVs with reduced power, bandwidth, and lower frequency. Experimental female USVs were unaffected [124].
	A1221 estradiol	P60	SD	10 min	For female rats, estradiol treatment decreased the number of step 50 kHz USVs following opposite-sex exposure. For male rats, A1221 treatment increased the number of rise and step 50 kHz USVs following opposite-sex exposure [125].
	A1221	P30–39	SD	5 min–4 h	PBCs affected the number of 50 kHz USVs for female rats but not male rats during same-sex play [126].
Diet and environmental stressors	High-fat diet	P7 P13	LE	10 min	Female pups on the high-fat diet produced more 1-sweep distress USVs, whereas male pups on the high-fat diet produced more 2-sweep distress USVs when compared to sex-matched controls [127].
	Maternal separation	P60	SD	15 min, 3 h	Brief maternal separation in pups resulted in changes in 22 kHz USVs in adulthood with fewer 22 kHz USVs in females but not in males, when compared to controls [75].
		P70–P90	SD	NS	After prenatal isolation, adult male rats produced 22 kHz USVs with greater duration compared to female rats [128].
		P120	LE	17 min	Maternal separation resulted in males producing more 22 kHz USVs in response to startle stimulus but did not affect female startle-induced 22 kHz USVs [129].
	Heat-induced convulsions	P10, P12	LE	2 min	Males produced more distress USVs (more category 5 and 6 USVs) compared to females [130].
	Moderate and extreme cold	P7–8	SD	70 min	Male pups produced more distress USVs than female pups; both sexes increased distress USVs in the presence of extreme cold temperature [131].
	Heat, light, and restraint stressors	P1 P6 P10 P14	SD	6 min	At P6, males produced more distress USVs than females during the first 2 min following maternal separation [132].
	corticotropin-releasing factor (CRF)	P6 P10 P14	SD	6 min	Overall, male pups produced more distress USVs than females [133].

A1221 = Aroclor 1221, AVP = arginine vasopressin, CRF = corticotropin-releasing factor, etOH = ethanol alcohol, LE = Long–Evans, MAM = methylazoxymethanol acetate, LPS = Liposaccharide, NS = not specified, P = postnatal day, PBCs = polychlorinated biphenyls, PCE = prenatal cocaine exposure, PD = Parkinson’s Disease, poly(I:C) = polyinosinic-polycytidylic acid, SD= Sprague–Dawley, SE = status epilepticus, SERT = serotonin transporter deficient, TMPP = trimethylolpropane phosphate, USV = ultrasonic vocalization, and VIN = vinclozolin.
